# Repeat cell-free DNA screening after initial false-positive results and term placental analysis for confined mosaicism: a prospective cohort study

**DOI:** 10.1007/s00404-025-08162-9

**Published:** 2025-08-31

**Authors:** Yvette C. Raymond, Shavi Fernando, Melody Menezes, Ben W. Mol, Tristan Hardy, Kara Levin, Emma Brown, Andrew McLennan, Daniel Lorber Rolnik

**Affiliations:** 1 Department of Obstetrics and Gynecology, Monash University, Clayton, Australia; 2 Monash Women’s, Monash Health, Clayton, Australia; 3 Monash Obstetrics, Clayton, Australia; 4 Victorian Clinical Genetic Services Ltd, Parkville, Australia; 5 Department of Pediatrics, The University of Melbourne, Melbourne, Australia; 6 Aberdeen Centre for Women’s Health Research, University of Aberdeen, Aberdeen, UK; 7 Repromed, Dulwich, Australia; 8 Monash IVF Group, Richmond, Australia; 9 Cytogenetics Laboratory, Monash Health Pathology, Clayton, Australia; 10 Sydney Ultrasound for Women, Sydney, Australia; 11 Faculty of Medicine and Health, The University of Sydney, Sydney, Australia; 12 Monash Ultrasound for Women, Melbourne, Australia

**Keywords:** Cell-free DNA, Non-invasive prenatal testing, Confined placental mosaicism, Placenta insufficiency

## Abstract

**Purpose:**

False-positive prenatal cell-free DNA screening (cfDNA) results may arise from confined placental mosaicism (CPM). This cohort study examines the persistence of high-risk cfDNA in the third pregnancy trimester after exclusion of fetal involvement, and the concordance of these results with CPM in the postpartum placenta.

**Methods:**

Pregnant individuals receiving a false-positive primary cfDNA result were recruited from Monash Health and Monash Ultrasound for Women in Melbourne, Australia, between August 2023 and December 2024. Participants underwent genome-wide repeat cfDNA screening (*r*-cfDNA) after 30 + 0 weeks’ gestation. Placental samples were collected and cytogenetically analysed postpartum.

**Results:**

This cohort included 21 individuals, of which 33.3% (7/21) screened high-risk on *r*-cfDNA. There was no significant association between *r*-cfDNA and CPM in the postpartum placenta (*p* = 0.397), as five (5/12, 41.7%) cases involving CPM were screened ‘low-risk’. Fetal-fraction was significantly lower (5.0% [IQR = 4.6–8.0%] vs. 9.0% [IQR = 7.0–13.5%], *p* = 0.025), and maternal BMI (Kg/m2) higher (29.0 [IQR = 25.7–31.2] vs. 23.3 [IQR = 22.0–24.9], *p* = 0.006), in false-positive cases not attributable to CPM detected by *r*-cfDNA or postpartum. High-risk *r*-cfDNA results were associated with smaller babies (median birthweight percentile = 12.3 [IQR = 4.4–21.7] vs. 31.9 [IQR = 21.5–55.5], *p* = 0.009), though the majority of outcomes were favorable.

**Conclusion:**

High-risk *r*-cfDNA is not a sensitive predictor of CPM, though high mosaic ratios are associated with adverse obstetric outcomes. An alternative explanation for false-positive cfDNA besides CPM may be low fetal fraction, particularly in the context of high maternal BMI.

## What does this study add to the clinical work

**Table:** 

One-third of verified ‘false-positive’ cell-free DNA (cfDNA) results remain high-risk after 30 weeks gestation, though cfDNA at this gestation is not a reliable predictor of confined placental mosaicism (CPM) in the term placenta. Results obtained with low-fetal fraction values determined to be ‘false-positive’ are less likely to represent CPM, and may instead be attributable to technical limitations of screening.	

## Introduction

Cell-free DNA (cfDNA) screening, colloquially known as non-invasive prenatal testing or NIPT, is a method of prenatal screening for fetal chromosome anomalies which utilizes cfDNA originating from the placental cytotrophoblast [1]. CfDNA is currently considered the most accurate prenatal screening method for trisomy 21 (T21), with higher sensitivity (99.7% vs. 92.1%) and lower false-positive rate (0.04% vs. 4.6%) compared to combined first-trimester screening (CFTS) [2, 3]. While original cfDNA panels screened for common autosomal trisomies (CATs; T21, trisomy 18 (T18) and trisomy 13 (T13)), and sex chromosome aneuploidies (SCAs), screening has more recently been expanded to genome-wide cfDNA (gwNIPT) [4]. This model encompasses rare autosomal trisomies (RATs) and segmental copy number variants (CNVs, sub-chromosomal deletions or duplications), with most platforms capable of detecting changes > 7 Mb.

The positive predictive value (PPV) of gwNIPT is highly variable, depending on the chromosome involved and the detected anomaly type, with PPV ranging from 85% for T21 [5] to 11% for RATs [6]. The most popular explanation for this discrepancy is confined placental mosaicism (CPM) [7]. As RATs are generally incompatible with pregnancy viability beyond 10 weeks’ gestation, these anomalies, when identified by cfDNA, are more likely attributable to CPM [8]. False-positive results for other anomalies, including CATs and SCAs, can also arise from CPM [9, 10].

There are several other contributors to false-positive cfDNA results besides CPM, including uterine fibroids, maternal mosaicism or malignancy, or a demised aneuploidy-affected twin. Although twin demise is usually considered exclusionary for cfDNA testing, it is frequently undetected [7]. Determining which pregnancies are affected by CPM following false-positive cfDNA is of clinical relevance, as studies have demonstrated evidence of association between CPM and other adverse obstetric outcomes, including fetal growth restriction (FGR), with the false-positive detection of certain chromosome anomalies such as trisomy 16 (T16) presenting particularly dire risk profiles [11].

The aim of this study was to assess the rate of screen-positive repeat cfDNA (*r*-cfDNA) in the third trimester amongst individuals with initial false-positive screening, and the concordance of these results with CPM in the term placenta. We also aimed to examine the obstetric outcomes associated with high-risk *r*-cfDNA results and CPM.

## Methods

This prospective cohort study was conducted in Melbourne, Australia, between August 2023 and December 2024. Participants were recruited from Monash Health (public hospital network comprising one tertiary and three secondary hospitals), and Monash Ultrasound for Women (private Fetal Medicine service provider with seven practices across Melbourne, Australia), after gaining informed written consent.

Eligible participants were those who had received a high-risk cfDNA result (with any platform) that was revealed to be ‘false-positive’ or discordant to the fetus by prenatal diagnostic investigations such as amniocentesis. Fetal uniparental disomy after cfDNA detection of trisomy was considered concordant, and thus such cases were excluded. Additional eligibility included age over 18 years and English speaking. A sample size of convenience was utilized due to the relative rarity of false-positive cfDNA in the general obstetric population and the novel descriptive nature of this study.

Recruited participants were invited to participate in a *r*-cfDNA screen after 30 + 0 weeks’ gestation, utilizing a whole-genome massively parallel sequencing platform (nest™, Monash IVF Group (Melbourne, Australia), Illumina, Inc. (San Diego, USA)). At delivery, four full-thickness placental biopsies sized 2 cm^2^ (one per quadrant) were extracted using the ‘human placenta tissue collection protocol’ published by the Stillbirth Centre of Research Excellence [12]. Samples were fixed in RPMI 1640 medium before three villi pieces were taken from each site, pooled and DNA extracted via standard extraction methodologies (EZ1, Qiagen, Venlo, The Netherlands). SNP Microarray was performed on the pooled DNA samples (Illumina Infinium GSA-24 v3.0, San Diego, USA), with genetic analysis restricted to the chromosome flagged by cfDNA (VIA, version 7.1, Bionano Genomics, San Diego, USA). The lower limit of mosaicism detection by SNP microarray has previously been determined to be 5–10% [13]. To prevent detection bias, the interpretation of the placental microarray was blinded to the results of *r*-cfDNA. For a small number of cases in which *r*-cfDNA was high-risk, but no anomalies were discovered in the postpartum placenta, a second *r*-cfDNA screen was offered 4–6 weeks’ postpartum. This was offered exclusively to ensure resolution of the high-risk result after clearance of the placenta, and to exclude a possible maternal source of the abnormal cfDNA, which would be expected to persist in the maternal circulation after birth.

The main outcomes of this study were the recurrence of high-risk *r*-cfDNA results in the third trimester and the concordance between these results and the term placental genome. Secondary outcomes included associations between screening variables and the likelihood of confirming CPM in the postpartum placenta. Pregnancy outcomes associated with both high- and low-risk *r*-cfDNA results, and CPM, were also examined.

Data were analyzed using Stata (StataCorp. 2021. Stata Statistical Software: Release 17. College Station), with *p*-values < 0.05 considered statistically significant. Association between categorical variables was assessed using Chi-squared and Fisher’s exact tests, and the Mann–Whitney *U* test for continuous variables. Analyses requiring adjustment for covariates (fetal fraction adjusted by maternal BMI, birthweight percentile adjusted by maternal height) were conducted using logistic and linear regression models providing normality of residuals was determined to protect against confounding [14, 15]. When assessing ‘adverse’ obstetric outcomes, classification included small for gestational age neonates (SGA, < 10th percentile), preterm birth, emergency cesarean birth, severe postpartum hemorrhage (PPH, > 1000 mL), pre-eclampsia, gestational diabetes mellitus (GDM), or stillbirth. Birthweight percentiles were calculated according to the Australian National chart by Dobbins et al. using gestational age and fetal sex [16].

Ethics approval for this research was obtained from the Monash Health Human Research Ethics Committee (ID 81975) and the Monash IVF Group Research, New Technologies and Science Communications Committee (approved 9th June 2023).

## Results

### Repeat cfDNA results

Our cohort included 21 individuals. The anomalies identified on primary cfDNA screening included seven CATs (four T13, two T18, one T21), five RATs (T3, T6, T8 and two T7), five CNVs (5q, 11q, 13q, 16p deletions, 12p duplication), and four SCAs (three X0, one XXY). All participants initially used cfDNA as a primary screening method, besides one X0 case associated with a nuchal translucency of 3.5 mm on first-trimester ultrasound. A summary of maternal and screening characteristics is provided in Table 1. The gestational window in which *r*-cfDNA was performed was between 30 + 5 and 34 + 6 weeks. The overall proportion of *r*-cfDNA results that returned as ‘high-risk’ in the third trimester was 7/21 (33.3%). This encompassed 2/7 (28.6%) CAT results (two T13), 3/5 (60.0%) RATs (one T3, two T7), 1/5 (20.0%) CNVs (12p duplication), and 1/4 (25.0%) SCAs (X0). There was no significant association between chromosome anomaly type and the frequency of high-risk *r*-cfDNA (*p* = 0.264).

**Table 1:** Maternal characteristics and screening variables, by repeat-cfDNA result

	Overall repeat cfDNA cohort	Low-risk repeat cfDNA	High-risk repeat cfDNA	*P-value for high-versus low-risk cohorts*
*Maternal characteristics*				
Maternal age (years), Median [IQR]	34.8 [33.2–36.9]	34.1 [31.9–36.9]	34.8 [34.2–38.2]	*0.360*
Maternal BMI (Kg/m^2^), Median [IQR]	25.5 [22.8–29.0]	25.9 [24.2–31.2]	23.0 [21.7–24.3]	*0.044*
IVF conception, n (%)	2 (9.5)	1 (7.1)	1 (14.3)	*0.567*
Uterine fibroids, n (%)	4 (19.1)	2 (14.3)	2 (28.6)	*0.407*
*Primary cfDNA screening*				
Gestation at initial cfDNA (days), Median [IQR]	77.0 [75.0–81.0]	76.5 [75.0–81.0]	80.0 [72.0–81.0]	*0.648*
Fetal fraction at initial cfDNA (%), Median [IQR]	8.0 [5.0–9.0]	6.8 [4.6–9.0]	9.0 [6.0–11.0]	*0.215* 0.918*
Frequency of anomaly type, n (%)				
CATs	7 (33.3)	5 (35.7)	2 (28.6)	*0.264*
RATs	5 (23.8)	2 (14.3)	3 (42.9)	
CNVs	5 (23.8)	4 (28.6)	1 (14.3)	
SCAs	4 (19.1)	3 (21.4)	1 (14.3)	
*Third-trimester repeat cfDNA*				
Gestation at repeat cfDNA, (days), median [IQR]	225.0 [221.0–229.0]	225.5 [223.0–230.0]	224.0 [220.0–229.0]	*0.454*
Fetal fraction on repeat cfDNA, (%), median [IQR]	23.0 [15.0–29.0]	19.5 [11.0–29.0]	25.0 [18.0–32.0]	*0.410* 0.691*
Mosaic ratio on repeat cfDNA, (%), median [IQR]	0.23 [0.02–0.58]	0.07 [< 0.01–0.18]	0.61 [0.33–0.86]	< *0.001*

*cfDNA* cell-free DNA, *BMI* body mass index, *IVF* in-vitro fertilisation, *CATs* common autosomal trisomies, *RATs* rate autosomal trisomies, *CNVs* copy number variants, *SCAs* sex chromosome aneuploidy

*adjusted for maternal BMI and gestation at cfDNA

### Placenta findings

**Fig. 1:**
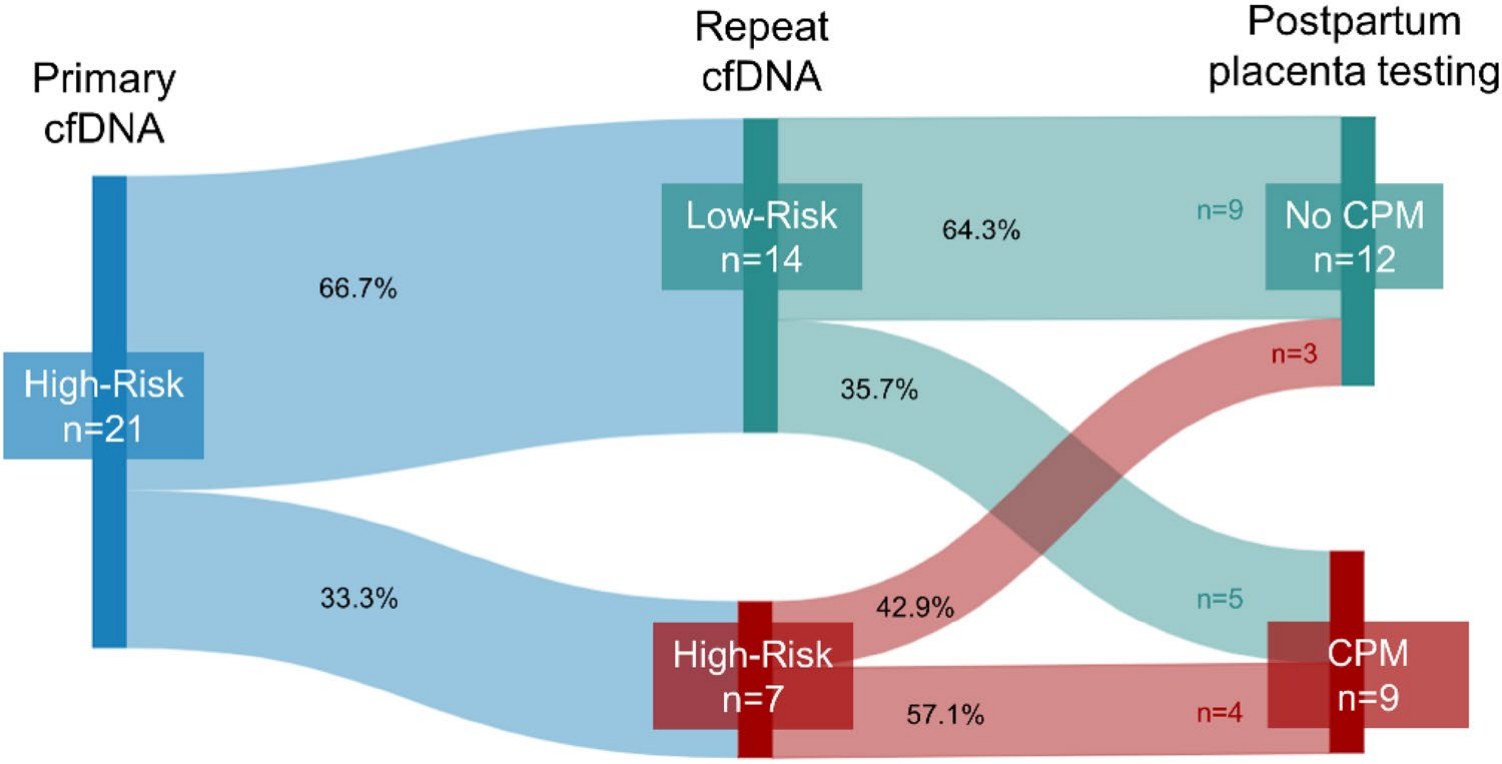
Progression of participants through repeat-cfDNA and postpartum placental testing. *cfDNA* cell-free DNA screening, *CPM* confined placental mosaicism

Placental specimens and obstetric outcomes were obtained for all participants. CPM was confirmed in 9/21 (42.9%) placentas; including 4/7 (57.1%) high-risk and 5/14 (35.7%) low-risk *r*-cfDNA results (Fig. 1). There was no significant association between high-risk *r*-cfDNA results and confirmation of CPM (odds ratio (OR) 2.40, 95% CI 0.41–14.11, *p* = 0.397). Maternal and screening characteristics of the cohort by CPM status are summarized in Table 2. Anomalies involved in CPM included one CAT (T18), four RATs (T3, T6, T7, T8), one CNV (12p duplication), and three SCA (three X0). All CPMs detected following high-risk *r*-cfDNA were concordant with the anomaly type indicated on screening (Table 3).

**Table 2:** Maternal characteristics and screening variables, by CPM status

	CPM			No CPM *N* = *9*	*P-value for combined CPM versus no-CPM cohorts (suspected CPM included)*	*P-value for confirmed CPM versus no-CPM cohorts (suspected CPM excluded)*
	Combined (confirmed + suspected) CPM* *N* = *12*	Confirmed CPM* *N* = *9*	Suspected CPM* *N* = *3*			
*Maternal characteristics*						
Maternal age (years), Median [IQR]	34.7 [33.3–36.0]	34.8 [33.2–35.9]	34.6 [34.2–38.2]	35.4 [32.1–37.0]	*0.754*	*0.508*
Maternal BMI (Kg/m^2^), Median [IQR]	23.3 [22.0–24.9]	23.0 [22.0–25.5]	23.5 [21.7–24.3]	29.0 [25.7–31.2]	*0.006*	*0.085*
Conception, n (%)	11 (91.7)	9 (100.0)	2 (66.7)	8 (88.9)	*0.686*	*0.314*
Spontaneous IVF	1 (8.3)	0 (0.0)	1 (33.3)	1 (11.1)		
Uterine fibroids, n (%)						
Not observed	10 (83.3)	8 (88.9)	2 (66.7)	7 (77.8)	*0.586*	*0.414*
Documented	2 (16.7)	1 (11.1)	1 (33.3)	2 (22.2)		
*Primary cfDNA screening*						
Gestation at initial cfDNA (days), median [IQR]	80.5 [72.5–83.0]	81 [77–85]	72.0 [70.0–81.0]	76.0 [75.0–77.0]	*0.336*	*0.078*
Fetal fraction at initial cfDNA (%), median [IQR]	9.0 [7.0–13.5]	9.0 [8.0–16.0]	8.0 [5.0–9.0]	5.0 [4.6–8.0]	*0.025* 0.309**	*0.023* 0.278**
Frequency of anomaly type, n (%)						
CATs	3 (25.0)	1 (11.1)	2 (66.7)	4 (44.4)	*0.017*	*0.017*
RATs	5 (41.7)	4 (44.4)	1 (33.3)	0 (0.0)		
CNVs	1 (8.3)	1 (11.1)	0 (0.0)	4 (44.4)		
SCAs	3 (25.0)	3 (33.3)	0 (0.0)	1 (11.1)		
*Third-trimester repeat cfDNA*						
Gestation at repeat cfDNA (days), median [IQR]	225.5 [222.5–229.5]	226.0 [225.0–229.0]	224.0 [215.0–237.0]	224.0 [219.0–226.0]	*0.411*	*0.253*
Fetal fraction at repeat cfDNA (%), median [IQR]	28.0 [21.5–32.0]	27.0 [23.0–32.0]	29.0 [12.0–32.0]	15.0 [11.0–19.0]	*0.016* 0.159**	*0.042* 0.184**
Mosaic ratio on repeat cfDNA, (%), median [IQR]	0.50 [0.28–0.76]	0.40 [0.23–0.59]	0.76 [0.33–0.86]	0.02 [0.0002–0.07]	*0.001*	*0.146*

*CPM* confined placental mosaicism, *cfDNA* cell-free DNA screening, *BMI* body mass index, *IVF* in-vitro fertilisation, *CATs* common autosomal trisomies, *RATs* rate autosomal trisomies, *CNVs* copy number variants, *SCAs* sex chromosome aneuploidy

*Suspected CPM denotes persistently high-risk repeat cfDNA in the third trimester, confirmed CPM confirmed confined placental mosaicism in the postpartum placental testing

**Adjusted for maternal body mass index and gestation at the respective cfDNA sampling

**Table 3:** Participant screening, placental and obstetric outcomes, summarised by individual case

N	Primary screening result (pre-enrolment)	Repeat-cfDNA result	Placenta finding	Birthweight percentile	Obstetric outcomes
1	T13–FF 5%	NAD–FF 11%	NAD	21.5	NAD
2	T13–FF 4.6%	NAD–FF 8%	NAD	40.1	NAD
3	T18–FF 11%	NAD–FF 33%	NAD	28.1	NAD
4	T21–FF 3%	NAD–FF 11%	NAD	90.0	GDM
5	XXY–FF 5.5%	NAD–FF 10%	NAD	21.5	GDM, emergency CS
6	5q deletion–FF 5%	NAD–FF 26%	NAD	55.5	NAD
7	11q deletion–FF 4%	NAD–FF 19%	NAD	47.1	NAD
8	13q deletion–FF 8%	NAD–FF 15%	NAD	62.6	GDM
9	16p deletion–FF 8%	NAD–FF 18%	NAD	35.7	GDM
10	T6–FF 8%	NAD–FF 27%	T6–15%	18.6	NAD
11	T8–FF 16%	NAD–FF 33%	T8–30%	95.7	Emergency CS, severe PPH (> 1000 mL)
12	T18–FF 4%	NAD–FF 29%	T18–20%	16.1	NAD
13	X0–FF 9%	NAD–FF 20%	X0–20%	25.7	NAD
14	X0–FF 18%*	NAD–FF 32%	X0–35%	23.4	NAD
15	T7–FF 8%	T7–FF 29%	NAD	1.7	NAD, FGR in context of short maternal stature and previous FGR pregnancy, postpartum cfDNA NAD
16	T13–FF 9%	T13–FF 12%	NAD	21.7	NAD, postpartum cfDNA NAD
17	T13–FF 5%	T13–FF 32%	NAD	34.6	GDM, postpartum cfDNA NAD
18	T3–FF 9%	T3–FF 18%	T3–15%	21.7	NAD
19	T7–FF 11%	T7–FF 25%	T7–10%	4.4	FGR
20	X0–FF 6%	X0–FF 23%	X0–40%	12.3	NAD
21	12p duplication–FF 17%	12p duplication–FF 33%	12p duplication	7.5	FGR

*cfDNA* cell-free DNA, *TX* Trisomy X, *FF* Fetal fraction, *NAD* no abnormalities detected, *TN* true-negative, *FN* false-negative, *FP* false-positive, *TP* true-positive, *GDM* gestational diabetes mellitus, *CS* caesarean section, *PPH* postpartum haemorrhage, *FGR* fetal growth restriction, *CPM* confined placental mosaicism

*cfDNA performed secondary to raised nuchal translucency (> 3 mm but < 5 mm) on first-trimester ultrasound

To assess for any predictive variables between cases of false-positive cfDNA with CPM, versus those likely attributable to other causes, we compared participants with low-risk *r*-cfDNA and no CPM findings (non-CPM), to the remainder of the cohort who received either a high-risk *r*-cfDNA, and/or were determined to have CPM postpartum. Non-CPM cases were associated with a significantly higher maternal BMI (29.0 [IQR = 25.7–31.2] Kg/m2), compared to the CPM cohort (pooled median 23.3 [IQR = 22.0–24.9] Kg/m2, *p* = 0.006). The fetal fraction on primary-cfDNA was lower amongst non-CPM cases (5.0% [IQR = 4.6–8.0%]) compared to the remainder of the cohort (9.0% [IQR = 7.0–13.5%], *p* = 0.025); however, this relationship was no longer significant after adjustment for maternal BMI (*p* = 0.092). The prevalence of fibroids amongst non-CPM cases (2/9, 22.2%) did not significantly differ from the pooled CPM cohort (2/12, 16.7%, *p* = 0.586), nor did gestation at cfDNA, either initial (76 [IQR = 75–77] vs. 80.5 [IQR = 72.5–83] days, *p* = 0.336) or at *r*-cfDNA (224 [IQR = 219–226] vs. 225.5 [IQR = 222.5–229.5] days, *p* = 0.411). Mosaic ratio values from primary-cfDNA were available for nine participants including five non-CPM cases, which showed no significant difference to the remaining cases (0.62 [IQR = 0.55–0.65] vs. 0.34 [IQR = 0.275–0.395], *p* = 0.191).

### Discordance between repeat-cfDNA and postpartum placenta

There were eight instances (38.1%) in which *r*-cfDNA was discordant with the term placenta (Table 3). These included three instances in which *r*-cfDNA was high-risk (two T13, one T7), but the high-risk result resolved postpartum, indicating clearance of the abnormal cfDNA after delivery. Subsequently, these results were attributed to low-level CPM missed by postpartum sampling. There was no significant distinction between *r*-cfDNA fetal fraction for these three cases, and cases flagged by *r*-cfDNA and confirmed in the placenta (n = 4) (29% [IQR = 12.0–32.0%] vs. 24% [IQR = 20.5–29.0%], p > 0.999), nor *r*-cfDNA mosaic ratio (0.76 [IQR = 0.33–0.86] vs. 0.56 [IQR = 0.39–0.79], *p* = 0.857).

In five cases, *r*-cfDNA returned low-risk, but CPM was discovered in the term placenta. These results involved T18, T6, T8 and two X0. Fetal fraction values did not differ significantly between these cases and CPMs detected by *r*-cfDNA (29.0% [IQR = 27.0–32.0%] vs. 24% [IQR = 20.5–29%], unadjusted *p* = 0.444, p adjusted by maternal BMI = 0.287). The mosaic ratios of these CPM cases reported as low-risk on *r*-cfDNA were notably lower than those of high-risk *r*-cfDNA (0.18 [IQR = 0.12–0.29] vs. 0.56 [IQR = 0.39–0.79]), though this relationship did not reach significance (*p* = 0.111).

### Obstetric outcomes

The median neonate birthweight percentile amongst high-risk *r*-cfDNA participants was 12.3% [IQR = 4.4–21.7%], compared to 31.9% [IQR = 21.5–55.5%] amongst those with low-risk *r*-cfDNA (unadjusted *p* = 0.009, p after adjustment for maternal height = 0.051). The median birthweight percentile was lower in pregnancies with confirmed CPM, compared to those with normal postpartum placenta findings (18.6% [IQR = 12.3–23.4%] vs. 35.1% [IQR = 21.6–51.3%], unadjusted *p* = 0.072, p adjusted by maternal height = 0.304). When the three instances involving high-risk *r*-cfDNA, which resolved postpartum were considered as CPM, these values shifted to 20.1% [IQR = 9.9–24.6%] versus 40.1% [IQR = 28.1–55.5%] (unadjusted *p* = 0.014, p adjusted by maternal height = 0.094).

There were no instances of preterm birth, pre-eclampsia, or stillbirth in the cohort. The total adverse obstetric event rate was 9/21 (42.9%). On excluding GDM, this rate was 5/21 (23.8%) (Table 3). This frequency did not significantly differ between high- and low-risk *r*-cfDNA results; adverse event rate including GDM = 4/7 (57.1%) versus 5/14 (35.7%, *p* = 0.319), or non-GDM event rate = 3/7 (42.9%) versus 1/14 (7.1%, *p* = 0.182). Comparing adverse outcomes between cases with and without CPM (when high-risk *r*-cfDNA cases with typical placenta biopsies were considered as CPM), the adverse event rates including and excluding GDM, respectively, were 5/12 (41.7%) versus 4/9 (44.4%, *p* = 0.623), and 4/12 (33.3%) versus 1/9 (11.1%, *p* = 0.258).

We found a significant association between *r*-cfDNA mosaic ratio and birthweight percentile, with percentile decreasing by 4.1% (95% CI 6.5–1.6%) for every 0.1 unit increase in mosaic ratio (p adjusted by maternal height = 0.003). Mosaic ratio was also higher amongst pregnancies with non-GDM adverse outcomes (0.76 [IQR = 0.61–0.97] vs. 0.10 [IQR = 0.01–0.31], *p* = 0.011).

## Discussion

### Main findings

In this cohort study, results of *r*-cfDNA testing in the third trimester were not significantly associated with CPM in the term placenta. While one third of participants screened high-risk again after 30 weeks’ gestation, CPM was confirmed, or suspected, in more than half of cases. The most common chromosomal anomalies involved in CPM were RAT and monosomy X. In pregnancies with low-risk *r*-cfDNA results and no CPM observed postpartum, maternal BMI was significantly higher. This is expected, as maternal body habitus is known to affect fetal fraction values and test accuracy, with increased mass diluting the proportion of circulating placental cfDNA, providing an alternative explanation for these false-positive results besides CPM [17].

There were three instances in which *r*-cfDNA returned as high-risk, but the postpartum placenta was unremarkable. These results were still attributed to CPM, as *r*-cfDNA repeated at a minimum of 4 weeks postpartum failed to identify any anomalies indicative of a maternal origin, which should theoretically be more easily identified after clearance of placental cfDNA [7]. Conversely, third-trimester *r*-cfDNA failed to detect CPM in five instances when it was discovered in postpartum tissue. This reduction in sensitivity for CPM compared to first-trimester cfDNA may be caused by the demise of the abnormal cell lineage by cell selection, resulting in dilution of the chromosome anomaly below the threshold of cfDNA detection with ongoing gestation and proliferation of euploid placental cells [18–20]. Collectively, both patterns of discordance between *r*-cfDNA and the term placenta suggest low-level mosaicism involved in cfDNA-detected CPM, especially in comparison to CPM detected by chorionic villus sampling [7].

Pregnancies that screened high-risk on *r*-cfDNA were associated with significantly lower neonatal birthweight percentiles compared to low-risk results, as were CPM cases, though these associations were no longer significant after adjusting for maternal height, albeit marginally. Adverse outcomes were also more frequent amongst high-risk *r*-cfDNA results, as well as CPMs, though these associations also did not reach significance. Finally, the mosaic ratio on *r*-cfDNA was inversely correlated with birthweight percentile, with an estimated reduction of four percentile points for every 10% increase in percentage mosaic ratio. Mosaic ratios were also significantly higher amongst pregnancies with adverse obstetric outcomes. Mosaic ratio quantifies the proportion of atypical cfDNA arising from the placenta, likely reflecting the extent of placental mosaicism. The extent of mosaicism is also known to correlate with the risk of placental insufficiency, with higher proportions generally incurring worse outcomes [21, 22].

### Clinical implications

CfDNA screening repeated in the third trimester is not a sensitive assessment of CPM, with more than half of cases confirmed postpartum (5/9) screened as low-risk. When false-positive results are obtained in the context of low fetal fraction, frequently coinciding with higher material BMI, CPM is less likely to be the causative agent. These results may instead be attributable to the technical limitations associated with less genetic material within the obtained plasma sample for analysis. Conversely, CPM is significantly more prevalent following high-risk RAT and monosomy *X* results.

The increased prevalence of adverse outcomes and reduced birthweight percentiles amongst pregnancies with CPM in this cohort gives support to the practice of fetal growth surveillance in cases of false-positive cfDNA, although, reassuringly, the majority of participant pregnancy outcomes were favorable. It is worth noting that all RATs in this cohort were mitotic-associated trisomies, as meiotic trisomies (trisomies 14, 15, 16, and 22) typically involve a higher proportion of mosaicism and are subsequently considered higher risk [7]. While adverse outcomes were more prevalent amongst high-risk *r*-cfDNA results in the third trimester, the benefits of discerning these pregnancies likely affected by CPM must be weighed against cost, and the likely psychological impact for parents receiving a second abnormal screening result [23]. Authors, therefore, do not support the use of *r*-cfDNA for clinical risk assessment.

### Comparison to prior research

To our knowledge, this is the first observational study involving repeat cfDNA in the third trimester. The frequency of CPM observed in this cohort was lower than the 67.0% found in the TRIDENT-1 and TRIDENT-2 studies, however, this is to be expected, as their cohort included a subset of pregnancies undergoing cfDNA after CFTS who were thereby at increased risk of CPM [24]. Our findings were also concordant with results of a 2022 meta-analysis investigating obstetric outcomes of cytogenetically-confirmed CPM (not including suspected CPM after cfDNA), which showed a three-fold increase in SGA neonates with CPM not involving trisomy 16. This adverse event rate was expectedly higher than in our cohort, as CVS was the primary method of identifying CPM in the meta-analysis, with abnormal CVS results generally involving higher degrees of placental mosaicism [20, 22]. Finally, the significant association between *r*-cfDNA mosaic ratio, fetal growth and pregnancy complications observed in our cohort was similar to that found by Eggenhuizen et al. in their 2024 study involving the TRIDENT-2 cohort, in which higher mosaic ratios were associated with SGA and hypertensive disorders. Importantly, these authors also acknowledge that mosaic ratio is not sufficient to clinically discern pregnancies likely affected by CPM from those at low risk [21].

### Strengths and limitations

As a whole placental analysis was not feasible in this study, so we utilized one biopsy from each placental quadrant. The primary limitation of this method is reduced sensitivity for low-level mosaicism. Notably, however, all cases also underwent *r*-cfDNA, thereby increasing the study’s detection capacity of low-level CPM, as demonstrated by the three instances in which CPM was considered after high-risk *r*-cfDNA but euploid placenta findings. Furthermore, as the level of placental mosaicism is associated with increased risk of placental insufficiency, we theorize that such low-level CPM going undetected by *r*-cfDNA and postpartum sampling is unlikely to be of clinical significance [7]. As false-positive cfDNA results are relatively rare events in the general obstetric population, we relied on a sample size of convenience of consecutive cases and were thereby underpowered in some analyses, namely the association between original cfDNA mosaic ratios and CPM, or adverse obstetric outcomes, after adjustment for maternal confounders [25]. The strengths of this study include its novel nature, as well as our 100% success rate in obtaining placenta samples for all recruited participants, which is uncommon in observational studies of this kind.

## Conclusion

After false-positive cfDNA results in the first trimester, third-trimester repeat testing remains high-risk in one-third of cases. However, persistently high-risk results do not reliably reflect placental mosaicism, with less than half of confirmed CPM cases being correctly identified by cfDNA in the third trimester. High-risk repeat results, however, particularly those with high mosaic ratio values, may be associated with adverse obstetric outcomes. CPM seems less common amongst primary results obtained with low fetal fraction values in the context of high maternal BMI, which provides an alternative explanation for test inaccuracy.

## Data Availability

No datasets were generated or analysed during the current study.
